# Detroit Preparatory School of Medicine

**Published:** 1865-12

**Authors:** 


Detroit Preparatory School of Medicine.—This effort to improve the standard of medical education we think will be well received by the profession; perhaps nothing is so much needed by students of medicine as the more familiar instruction of systematic private teaching. We understand the design to be, to give comprehensive and complete instruction upon elementary subjects, as preparatory to the advanced curriculum of the medical colleges. The teachers in this school are eminently practical men, and are supplied with abundant material for clinical observation by the Dispensary and Harper and St. Mary's Hospital. We believe that under their guidance this school will sustain the highest reputation for thorough and complete medical education.

